# Bachmann's Bundle Modification in Addition to Circumferential Pulmonary Vein Isolation for Atrial Fibrillation: A Novel Ablation Strategy

**DOI:** 10.1155/2023/2870188

**Published:** 2023-10-27

**Authors:** Jiaqi Sun, Sanbao Chen, Ming Liang, Qi Zhang, Ping Zhang, Mingyu Sun, Jian Ding, Zhiqing Jin, Yaling Han, Zulu Wang

**Affiliations:** Department of Cardiology, The General Hospital of Northern Theater Command, Shenyang, China

## Abstract

**Background:**

Bachmann's bundle (BB) is the main pathway of interatrial connection that could be involved in the development of atrial fibrillation (AF). Based on this hypothesis, we raised a novel ablation strategy, BB modification in addition to circumferential pulmonary vein isolation (CPVI-BB) in patients with AF.

**Methods:**

A retrospective cohort of patients with AF who underwent CPVI-BB or CPVI alone from March 2018 to July 2021 was enrolled in our study. Propensity score matching was performed in patients with paroxysmal AF and persistent AF, respectively, to reduce the risk of selection bias between the treatment strategies (CPVI-BB or CPVI alone). The primary endpoint was overall freedom from atrial arrhythmia recurrence through 12 months of follow-up.

**Results:**

Our propensity score-matched cohort included 82 patients with paroxysmal AF (CPVI group: *n* = 41; CPVI-BB group: *n* = 41) and 168 patients with persistent AF (CPVI group: *n* = 84; CPVI-BB group: *n* = 84). Among patients with persistent AF, one-year freedom from atrial arrhythmia recurrence rate was 83.3% in the CPVI-BB group and 70.2% in the CPVI group (log-rank *P* = 0.047). Among patients with paroxysmal AF, no significant difference was found in the primary endpoint between two groups (85.4% in the CPVI-BB group vs. 80.5% in the CPVI group; log-rank *P* = 0.581). In addition, procedure-related complications and recurrence of atrial tachycardia or atrial flutter were similar between the two treatment groups, regardless of the type of AF.

**Conclusions:**

BB modification in addition to CPVI is an effective approach in increasing the maintenance of sinus rhythm in patients with persistent AF, while it does not improve the clinical outcomes of radiofrequency catheter ablation in patients with paroxysmal AF.

## 1. Introduction

Catheter ablation is a well-established treatment for patients with symptomatic atrial fibrillation (AF) [1, 2]. Circumferential pulmonary vein isolation (CPVI) is identified as the cornerstone for ablation procedures due to the prominent role of the pulmonary vein (PV) in the initiation of AF [1, 2]. However, long-term outcomes of CPVI alone sometimes seem less satisfying, and additional linear ablation lesions have been investigated in paroxysmal and persistent AF in an attempt to improve ablation effectiveness [3–6].

Bachmann's bundle (BB) is a muscular structure connecting the right and left atrial walls, representing the main pathway of interatrial conduction. Recent trials have demonstrated the role of BB as a critical determinant of AF, and BB may be involved in the pathogenesis and perpetuation of a number of unstable reentry circuits [7–9]. Results of trials targeting the BB have been published, and in these trials, BB ablation through an epicardial approach and ablation in the region of BB insertion through an endocardial approach were effective in restoring sinus rhythm [5, 6, 10]. Therefore, we hypothesized that CPVI plus BB modification through an endocardial approach could improve clinical outcomes in AF.

## 2. Methods

### 2.1. Patient Population and Study Design

**Figure 1:**
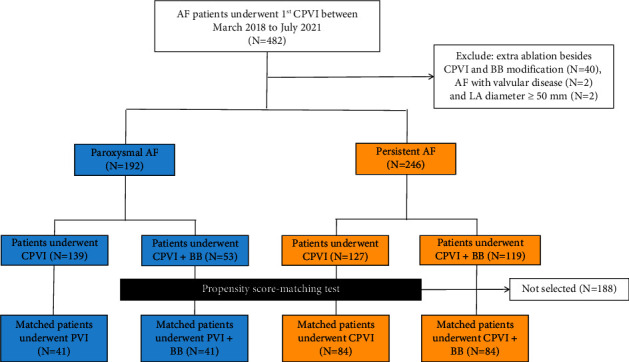
Flowchart of patients enrolled and included in the analyses. AF, atrial fibrillation; CPVI, circumference pulmonary vein isolation; BB, Bachmann's bundle.

A total of 482 consecutive patients undergoing their first radiofrequency (RF) ablation for AF from March 2018 to July 2021 were screened. Patients were excluded if (1) there was additional ablation besides CPVI and BB modification in the left atrium for an identified non-PV focus and/or poor atrial substrate; (2) AF with valvular disease; and (3) LA diameter ≥50 mm (Figure 1). The duration of persistent AF was assessed based on the documentation of AF on an electrocardiogram (ECG) or 24-h Holter ECG monitoring. In our institution, CPVI plus BB modification emerged as an alternative ablation method to CPVI and the decision to use BB modification was made the day before procedure to avoid selection bias during the procedure. Clinical data were extracted from the hospital's electronic health recording system, and propensity score models were constructed in patients with paroxysmal AF and persistent AF, respectively, to minimize selection bias in the baseline between the CPVI group and CPVI-BB group. The study protocol was carried out under the ethics principles established by the Declaration of Helsinki and approved by the local ethics committee. All patients provided written informed consent for the ablation procedure and enrollment in our study.

### 2.2. Common Pathways of the Procedure

In general, transesophageal echocardiography was routinely performed before procedures to evaluate the presence of thrombi. Antiarrhythmic drugs were stopped for at least 5 half-lives, and a single dose of a novel oral anticoagulant was skipped just before the procedure. An electrophysiological study and ablation procedure would be performed under conscious sedation with fentanyl. Briefly, two right femoral accesses (8.5-F sheaths) were used to perform transseptal puncture. Heparin was administered to maintain an activated clotting time of 300–350 seconds(s) during the procedure after transseptal puncture. A decapolar catheter was advanced to the coronary sinus through left femoral or right jugular access. A 20-mm decapolar circular or flexible mapping catheter was used to map the left atrium (Lasso or PentaRay; Biosense-webster, USA), and a 3.5-mm open irrigated-tip ablation catheter was used to deliver RF energy for the ablation. Before ablation, a detailed image reconstruction of the left atrium and PV was obtained, and the total area of the left atrium and area of the scar (voltage amplitudes of <0.15 mV) were calculated. RF energy was delivered at 35–40 W with a contact force of 5–15 g and an impedance of 120–140 ohms. The ablation index (AI), a lesion quality marker, was targeted at 360 to 380 for the posterior wall and 400 to 420 for the anterior wall and BB modification.

### 2.3. PVI and BB Modification Procedure

**Figure 2:**
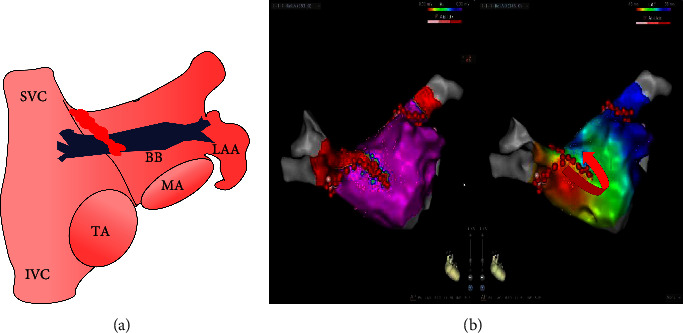
(a) Schematic diagram showing that BB modification was started from the roof of right superior PV toward the mitral annulus (12 o'clock direction in left anterior oblique view) ending at the level of the inferior base of left atrial appendage (LAA). Red tag represents ablation lesion. (b) Voltage and activation mapping performed after BB modification (CARTO picture/anteroposterior view). Voltage mapping (left) shows consecutive scar without gaps in the linear ablation lesions. Purple color represents the healthy area with an electrogram amplitude of ≥0.5 mV and red color represents voltage amplitudes of <0.15 mV. Activation mapping (right) shows significant conduction block along the linear ablation. SVC, superior vena cava; IVC, inferior vena cava; TA, tricuspid annulus; MA, mitral annulus; LAA, left atrial appendage; BB, Bachmann's bundle.

After the left atrial geometry was reconstructed, the right PV antrum was ablated first, followed by the left PV antrum in both groups. RF was applied 0.5–1.5 cm proximal to the ipsilateral PV ostia in a wide-area circumferential pattern. The Lasso or PentaRay catheter was placed in all 4 PVs to confirm PVI, and additional ablation would be performed if PVI was not complete. Isoproterenol was infused intravenously to elicit any nonpulmonary vein triggers. Persistent PVI was confirmed 30 minutes after the initial documentation of PVI. For patients in the CPVI-BB group, additional anterior linear ablation which originated from the roof of the right superior PV toward the mitral annulus (12 o'clock direction in left anterior oblique view) but was discontinued at the level of the inferior base of the left atrial appendage (LAA) was performed after CPVI. Different from conventional anterior wall linear ablation which connects the mitral annulus with the right superior PV [11], our ablation lesion is relatively short, just across the region of Bachmann's bundle (Figure 2). The endpoint of BB modification was the formation of consecutive scars without gaps in the linear ablation lesions and activation mapping to demonstrate an activation detour from one side to another of the line. If AF still existed after the completion of CPVI (CPVI plus BB modification in the CPVI-BB group), direct current cardioversion would be performed to restore sinus rhythm. To determine the effect of BB modification on LAA activation, a Lasso or PentaRay mapping catheter was positioned within LAA after BB modification. LAA electric isolation was defined as the disappearance of all LAA potentials and dissociation of LAA electric activity from the LA during LAA ectopics or pacing from LAA [12].

### 2.4. Patient Follow-Up and Clinical Outcomes

All patients were followed up at 3, 6, 9, and 12 months after the index procedure via telephone or hospital visit. An ECG was obtained at each follow-up point, and a 24-hour Holter ECG monitor was worn at the 3 and 12-month visits. In case of symptoms which suggested recurrent arrhythmia, an additional ECG or 24-hour Holter would be performed. All patients received antiarrhythmic drug (AAD) and anticoagulation for at least 3 months to avoid early recurrences and reduce the incidence of thromboembolic events. A proton pump inhibitor was given for at least 6 weeks. The primary outcome was freedom from clinical recurrence of atrial arrhythmias at 12 months after the index procedure. Recurrence of atrial arrhythmias was defined as any documented episode of AF or atrial tachycardia lasting at least 30 s [2]. Episodes of atrial arrhythmias within the first 3-month blanking period after the index procedure were considered early recurrence, and only recurrence after the blanking period was diagnosed as clinical recurrence. After the blanking period, AADs were prescribed in cases where AF/AT recurrence or frequent atrial premature beats were documented by ECG or 24-h Holter ECG monitoring. Other outcomes included procedure-related complications like groin hematoma, LAA electric isolation, pericardial effusion and tamponade, left phrenic nerve injury, atrial esophageal fistula, and other safety endpoints such as stroke and death.

### 2.5. Statistical Analysis

Continuous variables are expressed as means ± standard deviations or median with the interquartile ranges. Categorical variables are expressed as frequencies (*n*) and percentages (%). Continuous variables were compared using Student's *t*-test or Mann-Whitney *U* test. Categorical variables were compared using *χ*^2^ test or Fisher's exact test, as appropriate. To reduce the risk of selection bias between the treatment strategies (CPVI or CPVI-BB), propensity scores (PS) were estimated using a logistic regression model in patients with paroxysmal AF and persistent AF, respectively. 18 baseline covariates for persistent AF (all covariates in Table 1) and 16 baseline covariates for patients with paroxysmal AF (all covariates in Table 2) were included in the PS matching. Matching was done without replacement on the basis of logit-transformed propensity scores matching the nearest neighbor in a 1 : 1 fashion with a caliper of 0.02. The recurrence-free survival over follow-up was calculated using the Kaplan–Meier method, and survival distributions were compared by log-rank test. Statistical analysis was performed using IBM SPSS Statistics 25.0.

## 3. Results

From March 2018 to July 2021, 438 patients with AF (192 paroxysmal AF and 246 persistent AF) were enrolled in the present study. Among 192 patients with paroxysmal AF, 139 received only CPVI and 53 received additional BB modification. Among 246 patients with persistent AF, 127 received only CPVI and 119 received additional BB modification. Patient baseline characteristics are shown in Table 1 (persistent AF) and Table 2 (paroxysmal AF), respectively. After PS matching, the characteristics of patients on entry into the analysis were similar between CPVI groups and CPVI-BB groups, both in patients with paroxysmal AF and persistent AF.

**Figure 3:**
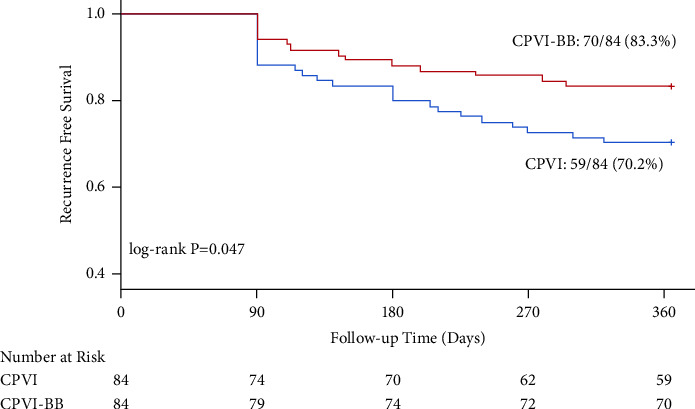
Kaplan–Meier analysis of freedom from recurrence of atrial arrhythmias in matched persistent AF cohort. CPVI, circumference pulmonary vein isolation; BB, Bachmann's bundle.

In the propensity score-matched persistent AF cohort, 1 pericardial effusion and 1 pseudoaneurysm occurred in the CPVI group, and 1 pericardial effusion occurred in the CPVI-BB group (*P* = 1.000). The Kaplan–Meier estimate of freedom from any atrial arrhythmia recurrence at 12 months was 83.3% in the CPVI-BB group, significantly higher than that in CPVI group (70.2%, Log-rank *P* = 0.047, Figure 3). No significant difference was found in the incidence of AFL or AT between the CPVI group and CPVI-BB group in patients with persistent AF (Table 3).

**Figure 4:**
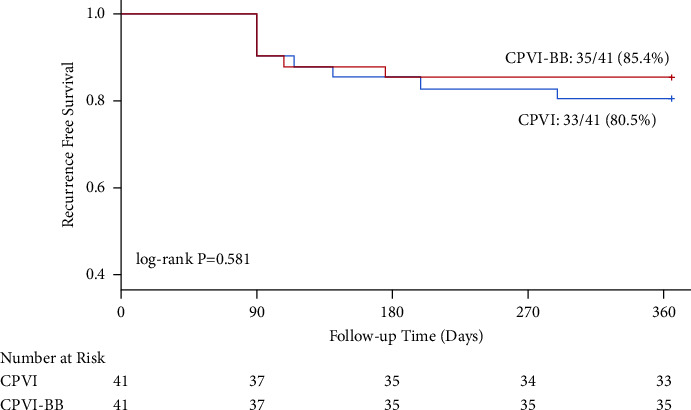
Kaplan–Meier analysis of freedom from recurrence of atrial arrhythmias in matched paroxysmal AF cohort. CPVI, circumference pulmonary vein isolation; BB, Bachmann's bundle.

In the propensity score-matched paroxysmal AF cohort, only 1 procedure-related complication, pericardial effusion, occurred in the CPVI-BB group. There was no difference in procedure-related complications between the two treatment groups (*P* = 1.000). At one-year follow-up, overall freedom of atrial arrhythmia recurrence was 80.5% in the CPVI group and 85.4% in CPVI-BB group (Log-rank *P* = 0.581, Figure 4). Additionally, the AFL or AT recurrent rate at 12-month follow-up was also similar (Table 4).

During the procedure, there were no procedure-related complications like LAA electric isolation, pericardial effusion and tamponade, or left phrenic nerve injury. Additionally, no stroke/transient ischemic attacks or deaths were observed in our overall cohort at one-year follow-up.

## 4. Discussion

### 4.1. Major Findings

In the current study, we investigated the safety and efficacy of circumferential pulmonary vein isolation with additional linear ablation targeting Bachmann's bundle in patients with AF. There are two key findings from this trial. First, compared with CPVI, BB modification in addition to CPVI was associated with a significant improvement in the maintenance of sinus rhythm for patients with persistent AF during the follow-up. However, as for patients with paroxysmal AF, the novel ablation approach did not improve the clinical outcome of RFCA. Second, BB modification did not increase the risk of procedure-related complications or iatrogenic arrhythmias caused by gaps in the ablation lines.

### 4.2. One-Year Clinical Outcomes of CPVI-BB in Patients with Persistent AF

The clinical outcome of patients with persistent AF is unsatisfying, and the optimal ablation approach is yet to be determined [13, 14]. It is noteworthy that BB modification in addition to CPVI results in 83.3% of patients with persistent AF being arrhythmia-free at one year, significantly higher than those who received CPVI only in our observations. Several recent trials investigating BB ablation in patients with persistent AF have produced similar results. De Martino et al. [5] described adjunctive BB ablation in the setting of hybrid ablation for long-standing persistent AF and found that one-year freedom from atrial arrhythmia recurrence rates in the BB group were superior to those without BB ablation. Similarly, substrate modification targeting the BB through an endocardial approach also acquires promising results [10]. Besides, other linear ablation strategies, which impaired Bachmann's bundle to some extent, such as “Dallas lesion” and the left atrial anterior linear lesion, proved effective in persistent AF [3, 15].

Recently, increasing evidence demonstrates the role of interatrial conduction, including Bachmann's bundle, coronary sinus conduction, and septal conduction in the maintenance of AF [16, 17]. Lim et al. [16] found that complete disconnection of the right atrium (RA) and LA resulted in an 80% chance of AF termination within 60 s in computer simulations. Kumagai et al. [18] observed that AF could not sustain itself unless a septal reentrant circuit involving the atrial septum existed. In some cases, unstable (short-lived) reentrant circuits of short cycle length in LA were transmitted to RA via BB before reentering LA via other interatrial conduction pathways, and thus these circuits were maintained in both atria. These circuits might disappear when conduction via BB is blocked, even if not completely.

### 4.3. One-Year Clinical Outcomes of CPVI-BB in Patients with Paroxysmal AF

During the follow-up, patients with paroxysmal AF who received additional BB modification were found not to be superior to those undergoing CPVI treatment, with a similar atrial arrhythmia recurrence at one year. Therefore, only 53 patients with paroxysmal AF received BB modification and BB modification were no longer performed on patients with paroxysmal AF. Different from persistent AF, the main mechanism of paroxysmal AF is regarded as triggered activity, predominantly originating in the PV antrum [19]. Non-PV trigger accounts for no more than 10% of PAF trigger [20]. In addition, CPVI not only eliminates triggers in the PV antrum but also affects the ganglionated plex or autonomic nerves [21]. Consistent with our observation, additional ablation such as CAFE-guided ablation, roof line, posterior box lesion, and Dallas lesion did not improve the clinical outcomes of patients with paroxysmal AF [20, 22, 23]. Since paroxysmal AF recurrence is usually caused by PV reconnection [24], permanent and complete CPVI is regarded as the cornerstone technique for the ablation of paroxysmal AF.

### 4.4. Procedure-Related Complications and Concern for Iatrogenic Arrhythmias

Although prior studies raised the concern that BB's ablation could impair the function of the left atrium appendage and result in a higher incidence of thromboembolic events by delaying electrical activation of LAA [11, 12], it is noteworthy that no LAA electric isolation and no complications specifically related to BB modification were found in our observation. Similar results were observed in other trials, in which BB were ablated through an epicardial or endocardial approach [5, 6]. BB is not the exclusive route of interatrial conduction, and wavefront propagates to LAA along other interatrial pathways when conduction across BB is impaired [9].

Besides concern for thromboembolic events, additional linear lesions, if incomplete, may be proarrhythmic [25]. In our study, no differences were found in the recurrence of atrial tachyarrhythmias, which differs from the results published by Sawhney et al. [25]. A major difference between our study and that published by Sawhney et al. is that the liner lesion in our ablation approach is relatively short. Short lines of conduction block result in short conduction time to propagate around linear lesions, and initiation of reentry through gaps in the linear lesions may become difficult [26].

### 4.5. Limitations

Several limitations should be acknowledged in the present study. First, this is a nonrandomized observational study, and the selection of the ablation approach was solely at the discretion of the operator. Although propensity score matching was performed to reduce selection bias, there may exist unmeasured confounding between treatment groups; what's more, patients with additional ablation for an identified non-PV focus and/or large area of low voltage in the left atrium were excluded in the present study, and our results may not be applicable for these people; as a result, our findings should be considered hypothetical and randomized controlled trials will be performed in the near future. Second, this study was performed at a single center and the external validity might be limited. However, overall freedom from atrial arrhythmias after CPVI treatment in patients with paroxysmal and persistent AF was consistent with those reported previously [13, 20, 27]. Finally, the recurrence of atrial arrhythmias was mainly based on ECG and scheduled 24-h Holter monitoring; short-lasting episodes of paroxysmal AF which caused no symptom may have been underdiagnosed.

## 5. Conclusions

Compared with CPVI alone, CPVI with additional BB modification is associated with better clinical outcomes in patients with persistent AF, while in patients with paroxysmal AF, the efficacy is comparable, suggesting that Bachmann's bundle may play an important role in the perpetuation of AF. Further clinical trials are warranted to confirm our promising results.

## Figures and Tables

**Table 1:** Baseline characteristics of patients with persistent AF.

Baseline characteristics	Nonmatched groups			Matched groups		
	CPVI (*n* = 127)	CPVI-BB (*n* = 119)	*P* value	CPVI (*n* = 84)	CPVI-BB (*n* = 84)	*P* value
Age, year	57.17 ± 9.53	57.48 ± 9.40	0.800	57.23 ± 9.45	57.64 ± 9.97	0.781
Male	98 (77.2%)	101 (84.9%)	0.124	70 (83.3%)	69 (82.1%)	0.838
BMI, kg/m^2^	26.38 ± 3.18	25.56 ± 3.16	0.042	26.09 ± 3.32	26.08 ± 3.26	0.994
Duration of AF	10 (2–23)	14 (5–24)	0.063	10 (2–20)	10 (4–24)	0.217
Long-standing AF	52 (41.9%)	60 (50.4%)	0.185	32 (38.1%)	38 (45.2%)	0.348
HBP	60 (47.2%)	50 (42.0%)	0.410	34 (40.5%)	42 (50.0%)	0.215
DM	21 (16.5%)	17 (14.3%)	0.626	11 (13.1%)	13 (15.5%)	0.659
CAD	20 (15.7%)	9 (7.6%)	0.047	11 (13.1%)	6 (7.1%)	0.201
History of stroke	17 (13.4%)	20 (16.8%)	0.453	13 (15.5%)	11 (13.1%)	0.659
Heart failure	14 (11.0%)	8 (6.7%)	0.237	6 (7.1%)	6 (7.1%)	1.000
Smoking	45 (35.4%)	49 (41.2%)	0.354	35 (41.7%)	36 (42.9%)	0.876
Drinking	36 (28.3%)	45 (37.8%)	0.114	32 (38.1%)	29 (34.5%)	0.630
CHA2DS2-VASc score	2 (2–3)	2 (1–3)	0.143	2 (1–3)	2 (1–3)	0.718
LVEF, %	56.83 ± 7.86	56.08 ± 7.54	0.451	56.63 ± 7.63	56.38 ± 7.28	0.828
LAD, mm	41.88 ± 4.54	42.26 ± 4.84	0.527	42.18 ± 4.98	42.29 ± 5.22	0.892
NT-pro BNP	714 (425–905)	834 (479–1004)	0.274	736 (383–900)	814 (530–987)	0.210
Total LA area, cm^2^	238.68 ± 38.35	244.10 ± 38.51	0.356	243.17 ± 40.24	245.08 ± 38.63	0.854
Scar area/total LA area, %	1.58 ± 1.06	1.84 ± 1.80	0.543	1.44 ± 1.22	1.76 ± 1.38	0.76

Values are reported as mean ± standard deviation or *n* (%). BMI: body mass index; DM: diabetes mellitus; CAD: coronary artery disease; LVEF: left ventricular ejection fraction; LAD: left atrium diameter; BNP: brain natriuretic peptide; LA: left atrial.

**Table 2:** Baseline characteristics of patients with paroxysmal AF.

Baseline characteristics	Nonmatched groups			Matched groups		
	CPVI (*n* = 139)	CPVI-BB (*n* = 53)	*P* value	CPVI (*n* = 41)	CPVI-BB (*n* = 41)	*P* value
Age, year	60.00 ± 9.91	60.79 ± 10.88	0.630	62.73 ± 7.71	60.80 ± 11.38	0.372
Male	82 (59.0%)	33 (62.3%)	0.679	22 (53.7%)	25 (61.0%)	0.503
BMI, kg/m^2^	25.09 ± 3.09	24.93 ± 2.65	0.753	25.10 ± 2.26	25.03 ± 2.45	0.899
Hypertension	64 (46.0%)	28 (52.8%)	0.400	18 (43.9%)	21 (51.2%)	0.507
DM	25 (18.0%)	20 (37.7%)	0.004	12 (29.3%)	12 (29.3%)	1.000
CAD	25 (18.5%)	14 (26.4%)	0.230	9 (22.0%)	10 (24.4%)	0.794
History of stroke	21 (15.1%)	14 (26.4%)	0.07	11 (26.8%)	10 (24.4%)	0.800
Heart failure	1 (0.7%)	2 (3.8%)	0.185	1 (2.4%)	1 (2.4%)	1.000
Smoking	51 (36.7%)	15 (28.3%)	0.274	11 (26.8%)	10 (24.4%)	0.800
Drinking	39 (28.1%)	16 (30.2%)	0.770	13 (31.7%)	10 (24.4%)	0.461
CHA2DS2-VASc score	2 (1–3)	2 (1–4)	0.053	2 (1–4)	2 (1–3)	0.899
LVEF, %	61.30 ± 4.71	61.04 ± 5.30	0.738	60.30 ± 4.80	61.4 ± 5.7	0.358
LAD, mm	36.53 ± 4.15	37.06 ± 5.02	0.462	37.40 ± 4.20	36.8 ± 5.0	0.549
NT-pro BNP	159 (68–341)	161 (68–344)	0.857	143 (78–468)	129 (68–344)	0.558
Total LA area, cm^2^	198.33 ± 28.67	204.34 ± 34.60	0.298	180.24 ± 33.12	186.97 ± 32.22	0.577
Scar area/total LA area, %	1.33 ± 1.98	1.42 ± 0.81	0.897	1.40 ± 1.62	1.39 ± 0.92	0.901

Values are reported as mean ± standard deviation or *n* (%). BMI: body mass index; DM: diabetes mellitus; CAD: coronary artery disease; LVEF: left ventricular ejection fraction; LAD: left atrium diameter; BNP: brain natriuretic peptide; LA: left atrial.

**Table 3:** Procedure results and clinical outcomes of patients with persistent AF after propensity score matching.

	Total (168)	CPVI (*n* = 84)	CPVI-BB (*n* = 84)	*P* value
Ablation time	86.53 ± 16.26	83.35 ± 14.77	89.18 ± 17.06	0.049
Procedure-related complications	3 (1.8%)	2 (2.4%)	1 (1.2%)	1.000
Clinical outcomes				
Clinical recurrence	39 (23.2%)	25 (29.8%)	14 (16.7%)	0.044
AFL or AT recurrence	3 (1.8%)	2 (2.4%)	1 (1.2%)	1.000
Early recurrence	37 (22.0%)	22 (26.2%)	15 (17.9%)	0.193
AAD after blanking period	46 (27.4%)	30 (35.7%)	16 (19.0%)	0.015

Values are reported as mean ± standard deviation or *n* (%). AF: atrial fibrillation; AFL: atrial flutter; AT: atrial tachyarrhythmias; AAD: antiarrhythmic drugs.

**Table 4:** Procedure results and clinical outcomes of patients with paroxysmal AF after propensity score matching.

	Total (*n* = 82)	CPVI (*n* = 41)	CPVI-BB (*n* = 41)	*P* value
Ablation time	77.28 ± 22.52	74.76 ± 22.20	79.80 ± 22.83	0.313
Procedure-related complications	1 (2.4%)	0	1 (2.4%)	1.000
Clinical outcomes				
Clinical recurrence	14 (17.1%)	8 (19.5%)	6 (14.6%)	0.557
AFL or AT recurrence	1 (1.2%)	1 (1.2%)	0	1.00
Early recurrence	14 (17.1%)	7 (17.1%)	7 (17.1%)	1.000
AAD after blanking period	13 (15.9%)	8 (19.5%)	5 (12.2%)	0.364

Values are reported as mean ± standard deviation or *n* (%). AF: atrial fibrillation; AFL: atrial flutter; AT: atrial tachyarrhythmias; AAD: antiarrhythmic drugs.

## Data Availability

Data used in this study are available upon reasonable request from the authors.
